# Nimotuzumab promotes radiosensitivity of EGFR-overexpression esophageal squamous cell carcinoma cells by upregulating IGFBP-3

**DOI:** 10.1186/1479-5876-10-249

**Published:** 2012-12-11

**Authors:** Lei Zhao, Li-Ru He, Mian Xi, Mu-Yan Cai, Jing-Xian Shen, Qiao-Qiao Li, Yi-Ji Liao, Dong Qian, Zi-Zhen Feng, Yi-Xin Zeng, Dan Xie, Meng-Zhong Liu

**Affiliations:** 1 Department of Radiation Oncology, Sun Yat-Sen University Cancer Center, No 651 Dongfeng Road East, Guangzhou 510060, China; 2 State Key Laboratory of Oncology in South China, Sun Yat-Sen University Cancer Center, No. 651 Dongfeng Road East, Guangzhou 510060, China; 3 Departments of Pathology, Sun Yat-Sen University Cancer Center, Guangzhou China; 4 Medical Imaging and Interventional Center, Sun Yat-Sen University Cancer Center, Guangzhou China

**Keywords:** EGFR, Esophageal squamous carcinoma cell, IGFBP-3, Nimotuzumab, Radiosensitivity

## Abstract

**Background:**

Epidermal growth factor receptor (EGFR) is suggested to predict the radiosensitivity and/or prognosis of human esophageal squamous cell carcinoma (ESCC). The objective of this study was to investigate the efficacy of Nimotuzumab (an anti-EGFR monoclonal antibody) on ESCC radiotherapy (RT) and underlying mechanisms.

**Methods:**

Nimotuzumab was administrated to 2 ESCC cell lines KYSE30 and TE-1 treated with RT. Cell growth, colony formation and apoptosis were used to measure anti-proliferation effects. The method of RNA interference was used to investigate the role of insulin-like growth factor binding protein-3 (IGFBP-3) in ESCC cells radiosensitivity treated with Nimotuzumab. *In vivo* effect of Nimotuzumab on ESCC radiotherapy was done using a mouse xenograft model.

**Results:**

Nimotuzumab enhanced radiation response of KYSE30 cells (with high EGFR expression) *in vitro*, as evidenced by increased radiation-inhibited cell growth and colony formation and radiation-mediated apoptosis. Mechanism study revealed that Nimotuzumab inhibited phosphorylated EGFR (p-EGFR) induced by EGF in KYSE30 cells. In addition, knockdown of IGFBP-3 by short hairpin RNA significantly reduced KYSE30 cells radiosensitivity (*P*<0.05), and even after the administration of Nimotuzumab, the RT response of IGFBP-3 silenced KYSE30 cells was not enhanced (*P*>0.05). In KYSE30 cell xenografts, Nimotuzumab combined with radiation led to significant tumor growth delay, compared with that of radiation alone (*P*=0.029), and also with IGFBP-3 up-regulation in tumor tissue.

**Conclusions:**

Nimotuzumab could enhance the RT effect of ESCC cells with a functional active EGFR pathway. In particular, the increased ESCC radiosensitivity by Nimotuzumab might be dependent on the up-regulation of IGFBP-3 through EGFR-dependent pathway.

## Introduction

Esophageal cancer is an aggressive cancer constituting the sixth cause of cancer-related deaths worldwide [1]. The main histological type is squamous cell carcinoma (SCC) in East Asian. Despite ongoing research in the treatment of ESCC, the prognosis for patient long-term survival remains poor. Surgery alone for locally advanced disease results in a 5-year survival rate of only 20-25%. Although the addition of combined modality strategies of pre- or post-operative chemoradiotherapy is reported to improve the 5-year survival rates of 10-15% [2,3], the therapeutic toxicity remains to be a big problem to overcome. Therefore, an efficacious therapy with minimal toxicity is still urgently needed. Along with a better understanding of molecular pathways of esophageal carcinogenesis, the focus of recent study has shifted toward testing newer agents that target specific molecular abnormalities known to occur in ESCC.

In preclinical studies involving various cancer models, including ESCC, overexpression of EGFR has been found to contribute to epithelial cell proliferation, differentiation, and migration [4,5] and had an inverse relationship to tumor radiocurability [6,7]. In addition, overexpression of EGFR has been shown to correlate with lower tumor control rates after irradiation in several studies [8,9]. Recently, combination of radiotherapy and EGFR inhibitors was reported to improve local tumor control compared to irradiation alone, but it is also true that conflicting results exist [10,11].

Nimotuzumab is a humanized anti-EGFR monoclonal antibody (mAb) that binds to the extracellular domain of the EGFR and inhibits EGF binding [12]. In a preclinical study, Nimotuzumab demonstrated marked antiproliferative, proapoptotic, and antiangiogenic effects in tumors that overexpress EGFR [13]. Now, Nimotuzumab has been approved in several countries for the treatment of head and neck tumors [14] and glioma [15], and is in clinical trials for various tumor types including head and neck tumors, colorectal, glioma (pediatric and adult), pancreatic, prostate, non-small cell lung, cervical, and breast cancer [14-17]. And clinically, Nimotuzumab has shown high safety and low toxicity without severe skin and mucosa toxicities commonly associated with other EGFR targeting antibodies [18]. In esophageal cancer, EGFR overexpression by immunohistochemistry or gene amplification by fluorescent in situ hybridization (FISH) analysis occurs in 30-90% of tumors. Generally, EGFR overexpression is more common in ESCC than in esophageal adenocarcinoma [19]. In our previous study, we found that Nimotuzumab could increase ESCC chemosensitivity to DDP by upregulating IGFBP-3 expression through EGFR-dependent pathway *in vitro*[20]. However, whether or not Nimotuzumab can radiosensitize ESCC both *in vitro* and *in vivo* with different expression levels of EGFR is unknown. In the present study, we therefore investigated the efficacy of the anti-epidermal EGFR mAb, Nimotuzumab, on ESCC cells radiotherapy and its potential underlying mechanisms *in vitro* and *in vivo*.

## Methods

### Cell lines and reagents

The human ESCC cell lines, KYSE30 and TE-1, were obtained from the Cell Bank of the Chinese Academy of Sciences (Shanghai, China). Both cell lines were cultured in RPMI 1640 medium (Invitrogen, Carlsbad, CA) supplemented with 10% fetal bovine serum (Bioind, Kibbutz Beit, Israel) in a humidified 5% CO_2_ atmosphere at 37°C. In previous study, we have confirmed there is a higher level of EGFR protein expression in KYSE30 cells compared with that in TE-1 cells at baseline by Western blot and immunocytochemical staining analysis [20]. Nimotuzumab was provided by Biotech Pharmaceuticals Co., Ltd. (Beijing, China). All the other chemical reagents and solvent were from Sigma-Aldrich (St. Louis, MO).

### Measurement of cell toxicity

KYSE30 and TE-1 cells were plated in 96-well plates at a density of 2,000-3,000 cells/well and incubated for 72 hours with indicated concentrations (6.25 μg/mL, 12.5 μg/mL, 25 μg/mL, 50 μg/mL, 100 μg/mL, 200 μg/mL) of Nimotuzumab alone or combined with various doses of X-ray with a 160 KV X-irradiator (Rad Source Technologies, Inc. Suwanee, GA) at a rate of approximately 8.7 Gy/minute. The cells were then incubated for 4 hours in medium containing 3-[4,5-dimethylthiazol-2-yl]-2,5-diphenyltetrazolium bromide (MTT) and lysed in dimethylsulphoxide (DMSO). This procedure was performed as previously described [21]. The conversion of MTT to formazan by metabolically viable cells was monitored using a 96-well microtiter plate reader at an absorbance of 570 nm.

### Clonogenic assay

Cells were supplemented with 1% fetal bovine serum in the absence or presence of Nimotuzumab. After incubation for 24 hours, the cells were exposed to various doses of X-ray. The cells were cultured in drug-free medium with 10% fetal bovine serum for 10–14 days, then fixed and stained with crystal violet and counted manually. Colonies containing more than 50 cells were counted. The surviving fractions were determined as ratios of the plating efficiencies (PE = counted colonies/seeded cells × 100) of the irradiated cells to the non-irradiated cells. The DMF_10_ was established to analyze the effects of Nimotuzumab on the radiosensitivity of ESCC cells (DMF_10_ = radiation dose untreated cells/radiation dose treated cells). To fit the data a linear quadratic model lnS = −(α*D* + β*D*^2^) was applied, using origin 7.5.

### Western blot analysis

This procedure has been previously described [20,22]. The cells were lysed using a 50 nM Tris (pH7.5), 150 mM NaCl, and 0.5% NP-40 solution on ice. Fifty micrograms of total protein from each sample was resolved on a 12% bis–tris gel with MOPs running buffer and transferred to nitrocellulose membranes. The blots were probed with various antibodies, including anti-EGFR, p-EGFR-1068 and Caspase-3 (Cell Signaling Technology, Beverly, MA), IGFBP-3 (Santa Cruz Biotech, CA).

### Apoptosis assays

The ESCC cells undergoing apoptosis were distinguished from live and necrotic cells by the use of annexin-V and propidium iodide (PI) staining using Apoptosis Detection Kit (Invitrogen). Briefly, ESCC cells were treated with Nimotuzumab (100 μg/mL, 24 hours) and/or irradiated with either 2Gy or 10Gy, and harvested after 48 hours post-irradiation. Aliquots of 10^5^ cells were incubated with annexin-V/PI for 15 minutes at room temperature. Cells were then analyzed by means of flow cytometry using a two-color fluorescence activated cell sorting (FACS) analysis (Beckman Coulter, cytomics FC 500, CA).

### Knocking down of IGFBP-3 by lentiviral short hairpin RNA (shRNA)

A previously constructed lentivirus transfer vector, which encoded shRNA targeting IGFBP-3 and can efficiently silence endogenous IGFBP-3 in cancer cells was utilized [23]. Briefly, lentiviral pGIPZ vectors carrying shRNA directed against human IGFBP-3 (V2LHS_111628 and 225584) and a non-silencing scramble control sequence (RHS4346) along with green fluorescent protein (Open Biosystems, Huntsville, AL) were used. Three days after Lentiviral pGIPZ vectors infection, the ESCC cells were passaged and selected with geneticin (400 μg/ml; Invitrogen, Carlsbad, CA) for 3 weeks for resistant colonies. Geneticin-resistant clones were harvested and subjected to Western blot analysis to examine the knocking down efficiency of IGFBP-3 by lentivirus shRNA.

### Xenograft models and therapy

Animal experiments were conducted in accordance with “Guidelines for the Welfare of Animals in Experimental Neoplasia”. KYSE30, 3×10^6^ or TE-1, 1×10^7^ cells were injected subcutaneously in the right hind limbs of female BALB/c nude mice (4–5 weeks old). Treatment was initiated when tumors in each group achieved an average volume of approximately 170-200 mm^3^ (about 10–14 days). And then the mice were randomized into the following three groups: control, radiation alone (8Gy × 1), and Nimotuzumab (a single dose of 1.0 mg per mouse) combined with radiation(N = 6 per group). Mice in the control and radiation alone groups were injected with physiological saline. Radiation began 6 hours after drug treatment. After 4 weeks, mice were sacrificed and tumors were resected and weighed. Tumors were measured at regular 3-days intervals and tumor volume was determined by caliper measurement of tumor length (L) and width (W) according to the formula LW^2^/2.

### Immunohistochemical analysis

This procedure was performed as previously described [24]. The tumors slides of xenograft were deparaffinized in xylene, rehydrated through graded alcohol, immersed in 3% hydrogen peroxide for 10 minutes to block endogenous peroxidase activity, and antigen retrieved by pressure cooking for 3 minutes in citrate buffer (pH = 6). To block nonspecific binding, the slides were pre-incubated with 10% normal goat serum at room temperature for 30 minutes. Subsequently, the slides were incubated overnight at 4°C in a moist chamber with mouse monoclonal antibody anti-p-EGFR (Cell Signaling Technology; 1:1600 dilution) and rabbit polyclonal antibody anti-IGFBP-3 (Santa Cruz Biotechnology; 1:100 dilution). The slides were sequentially incubated with a secondary antibody (Envision; Dako, Glostrup, Denmark) for 1 hour at room temperature, and stained with DAB (3,3-diaminobenzidine). Finally, the sections were counterstained with Mayer’s hematoxylin, dehydrated and mounted. A negative control was obtained by replacing the primary antibody with a normal murine or rabbit IgG. Known immunostaining-positive slides were used as positive controls.

### Statistical analysis

All descriptive statistics, including mean ± SD, were performed. Unpaired Student’s *t*-test was used to evaluate differences between the control group and each treatment group in all *in vitro* and *in vivo* studies performed, with the resultant *P* value representing a two-sided test of statistical significance. SPSS 13.0 software (SPSS, Inc., Chicago, IL) was used for statistical analyses with a *P* value of < 0.05 considered statistically significant.

## Results

### Nimotuzumab enhanced radiation response of ESCC cells with high expression of EGFR *in vitro*

**Figure 1:**
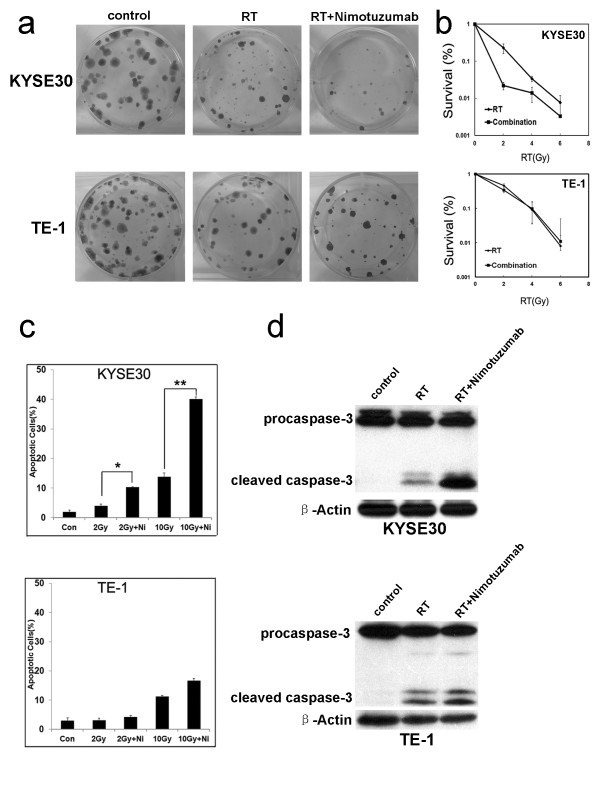
**Nimotuzumab enhanced the sensitivities of EGFR overexpressed ESCC cells to RT *in vitro. ***(**a **and **b**) KYSE30 and TE-1 cells were incubated with or without Nimotuzumab (100 μg/mL) for 24 hours, and exposed to the indicated doses of RT. The colonies were stained and counted, and survival curves were constructed from three independent experiments. N = 3, bars, ± SD. The result is representative at 6 Gy of RT dosage. (**c**) KYSE30 and TE-1 cells were treated with RT (2 Gy, 10 Gy) in presence or absence of Nimotuzumab (100 μg/mL) for 48 hours. The control, RT and combination treatment groups were collected for staining with annexin-V/PI. The samples with a statistically significant difference compared with the groups as indicated (N = 3, bars, ± SD.) (**d**) Cell lysates were subjected to immunoblot with caspase-3-specific antibody for cleavage of caspase-3.

Firstly, we examined weather Nimotuzumab has chemotherapeutic-induced toxic effects in ESCC cells *in vitro*. We found that Nimotuzumab was not toxic to either KYSE30 or TE-1 cell line, even when they were treated with 200 μg/mL of Nimotuzumab for 72 hours (*P* > 0.05), as determined by the MTT assay (Additional file 1: Figure S1). Next, we tested whether or not Nimotuzumab enhanced ESCC cells sensitivity to radiotherapy *in vitro* by MTT assay. KYSE30 and TE-1 cells were treated with various doses of RT in the presence of different concentrations of Nimotuzumab. The results showed that compared to treatment with RT alone, combination of RT and Nimotuzumab resulted in a significant higher level of cell death in KYSE30 cells (*P* = 0.01; Additional file 2: Figure S2A), but not significant in TE-1 cells (the cell line with low levels of EGFR, *P* = 0.087; see Additional file 2: Figure S2B). Further clonogenic assays confirmed the above results, in which the DMF_10_ (dose-modifying factor at a 10% survival level) presented in the experiment were 1.68 (*P* = 0.01) for the KYSE30, whereas Nimotuzumab had no effect on radiosensitivity of TE-1 cells (DMF_10_ was 0.98, *P* = 0.2) (Figure 1a and b).

In our study, the levels of cell apoptosis were further examined in ESCC cells treated with RT before and after Nimotuzumab treatment. In KYSE30 cells, we observed that the percentage of positive cells for annexin-V/PI significantly increased in combination treatment group as compared to that in RT alone group (Figure 1c and Additional file 3: Figure S3A). However, the similar result was not observed in TE-1 cells (Figure 1c and Additional file 3: Figure S3B). In addition, we examined apoptosis related caspase-3 activation in KYSE30 cells. We found that caspase-3 was further activated in 72 hours after exposure to Nimotuzumab and RT (Figure 1d).

### Nimotuzumab inhibited EGFR phosphorylation in EGFR overexpressing ESCC cells

**Figure 2:**
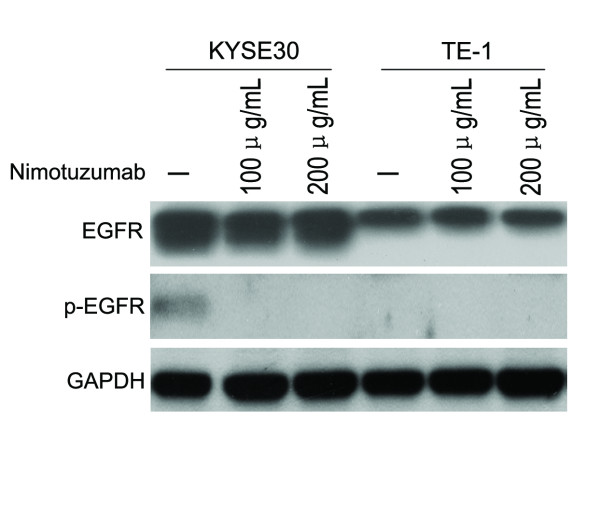
**Modulation of EGFR and p-EGFR expression in ESCC cells by Nimotuzumab. **Both KYSE30 and TE-1 cell lines were cultured with 0, 100 and 200 μg/mL Nimotuzumab for 72 hours in medium containing 10% FBS. Western blot analysis was performed to examine the expression levels of EGFR and p-EGFR. Nimotuzumab did not influence the levels of EGFR in both cells, but it down-regulated the expression of p-EGFR in KYSE30 cells with overexpression of EGFR. Representative result was shown of triplicate experiments.

To investigate potential mechanisms of Nimotuzumab in the promotion of ESCC cells radiosensitivity, we first studied whether or not Nimotuzumab could influence the levels of EGFR in ESCC cells. Our results showed that in the levels of EGFR under identical culture condition (with 10% fetal bovine serum), Nimotuzumab did not influence the total levels of EGFR in either EGFR overexpressing KYSE30 or EGFR low-expressing TE-1 cell line. However, Nimotuzumab did down-regulate the levels of p-EGFR in KYSE30 cells. In TE-1 cells, owing to the presence of p-EGFR is not detectable in the control cells (without adding Nimotuzumab), the effect of p-EGFR downregulation cannot be observed in cells treated with Nimotuzumab (Figure 2). We hypothesized that the multiple low-concentration cytokines in fetal bovine serum could only activate EGFR pathway in EGFR high-expression KYSE30 cells, however, this effect was not observed in TE-1 cells due to low-expression EGFR. These results revealed that Nimotuzumab down-regulated the levels of EGFR phosphorylation in EGFR overexpressing ESCC cells.

### IGFBP-3 up-regulation is involved in nimotuzumab-enhanced radiosensitivity of ESCC cells

**Figure 3:**
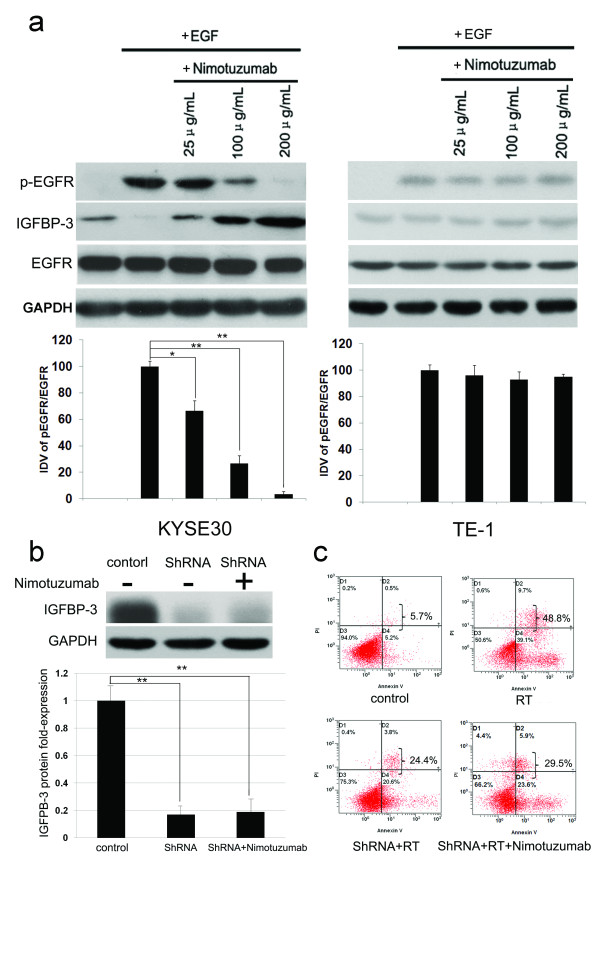
**The levels of IGFBP-3 are responsible to Nimotuzumab-enhanced radiosensitivity of EGFR overexpressing ESCC cells. **(**a**) KYSE30 and TE-1 cells were deprived of serum overnight and then incubated first for 3 hours with or without Nimotuzumab and then for an additional 15 minutes in the additional absence or presence of EGF (100 ng/mL). Cell lysates were evaluated for IGFBP-3, phosphorylated and total EGFR by Western blot. (**b**) IGFBP-3 silenced KYSE30 cells were harvested after 72 hours exposure to Nimotuzumab (100 μg/mL) or not. The expression levels of IGFBP-3 in control (non-silenced cells), IGFBP-3-silenced and Nimotuzumab-treated IGFBP-3-silenced cells were determined by Western blot. The experiments were performed in triplicate. (**c**) IGFBP-3-silenced KYSE30 cells were treated with RT (10 Gy) in presence or absence of Nimotuzumab (100 μg/mL) for 72 hours. All cells were collected for staining with annexin-V/PI, and cell death was measured by flow cytometry. In RT experiment, the radiosensitivity of KYSE30 cells was significantly inhibited after IGFBP-3 silence, and the cell death rates were reduced from 46.2 ± 2.1% to 25.9 ± 3.1% (*P* < 0.05). After the administration of Nimotuzumab, the RT response of IGFBP-3-silenced KYSE30 cells was not enhanced (the cell death rate was 28.6 ± 2.8%) (*P* > 0.05). The experiments were performed in triplicate.

Recent studies documented that IGFBP-3, one of EGFR downstream targets, plays an important role in modulation of radiosensitivity of different human cancers [25], and EGF could down-regulate IGFBP-3 expression in esophageal squamous carcinoma cells [26,27]. We therefore investigated whether Nimotuzumab inhibited EGF-induced EGFR phosphorylation, and consequently down-regulated IGFBP-3 in ESCC cells. To investigate the effect of EGF on ESCC cells, KYSE30 and TE-1 cells were deprived of serum overnight. As demonstrated in Figure 3a, EGFR phosphorylation could not be detected without the addition of EGF. Nimotuzumab inhibited the levels of EGFR phosphorylation induced by EGF (100 ng/mL) in a dose-dependent manner, and concurrent with an up-regulated expression of IGFBP-3 in EGFR overexpressing KYSE30 cells (Figure 3a, *left*). But this effect was not observed in EGFR low-expressing TE-1 cells (Figure 3a, *right*). To further investigate the effect of IGFBP-3 in the process of Nimotuzumab modulating of ESCC cells radiosensitivity, the expression levels of IGFBP-3 in KYSE30 cell line were first knocked down by a specific shRNA targeting *IGFBP-3* gene (Figure 3b). Next, the IGFBP-3-silenced and non-silenced KYSE30 cells were exposed to Nimotuzumab (100 μg/mL), 10Gy X-irradiation or combination, and maintained for 72 hours in culture. Apoptosis and clonogenic assay demonstrated that compared to that in non-silenced KYSE30 cells, the radiosensitivity in IGFBP-3-silenced KYSE30 cells were significantly inhibited (Figure 3c and Additional file 4: Figure S4A), and furthermore, even after the administration of Nimotuzumab, the radiotherapy response of IGFBP-3 silenced KYSE30 cells was not enhanced (*P* > 0.05, Figure 3c and Additional file 4: Figure S4B). These results provided evidences that the level of IGFBP-3 is responsible, at least partly, to the enhanced radiosensitivity by Nimotuzumab in ESCC cells with overexpression of EGFR.

### Nimotuzumab increased IGFBP-3 expression and radiosensitivity of ESCC cells with high expression of EGFR *in vivo*

**Figure 4:**
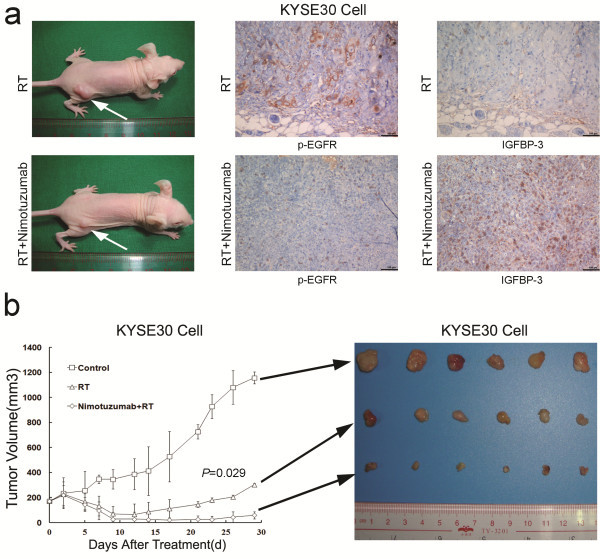
**Effects of Nimotuzumab on the response of KYSE30 ESCC xenografts to RT. **(**a**) Representative KYSE30 cell xenografts in RT treatment and RT + Nimotuzumab treatment groups. p-EGFR and IGFBP-3 expressions of KYSE30 cell xenograft tumors were examined by IHC. p-EGFR was positively stained in the majority of RT-treated KYSE30 cells of xenograft, while most cells had negative expression of IGFBP-3. In RT + Nimotuzumab-treated KYSE30 cell xenograft tumor, most tumor cells showed negative expression of pEGFR, while the majority of cells was positively stained with IGFBP-3 (100×). (**b**) KYSE30 cells were injected subcutaneously in athymic nude mice. Treatment was initiated when tumors in each group (N = 6) achieved an average volume of approximately 170–200 mm^3^. The mice were randomized into three groups: control, radiation alone (8Gy × 1), and Nimotuzumab (a single dose of 1.0 mg per mouse) combined with radiation. Tumor volume was determined at the indicated time points thereafter.

To test the effects of Nimotuzumab on ESCC cells radiosensitivity *in vivo*, we further constructed ESCC xenograft models by injecting tumor cells subcutaneously in BALB/c nude mice. *In vitro*, radiotherapy alone did not induce up-regulation of IGFBP-3 expression in KYSE30 cells (Additional file 5: Figure S5A). As shown in Figure 4a, expression levels of p-EGFR and IGFBP-3 in tumors slides of KYSE30 cell xenografts after RT with or without Nimotuzumab treatment were examined using immunohistochemical analysis. Expression of p-EGFR and IGFBP-3 in tumors was quantified by counting positive cells in 3 fields from 3 mice. Same as the results *in vitro*, after Nimotuzumab administration, the level of EGFR phosphorylation was inhibited in KYSE30 cell xenograft tumors, concurrent with IGFBP-3 up-regulation. In KYSE30 xenografts of nude mice, there was a significant increase in tumor growth delay in animals treated with combination of Nimotuzumab and RT, compared with that treated with radiation alone (*P* = 0.029; Figure 4b). However, in TE-1 xenografts, we observed that tumor growth was also inhibited by radiation, but this radiotherapy effect could not be enhanced by concurrent treatment of Nimotuzumab (*P* = 0.672; Additional file 5: Figure S5B).

## Discussion

Clinically, RT is one of the most important therapeutic methods for ESCC, especially for those unresectable ones. Thus, new strategies that could enhance ESCC RT response and have no further therapeutic toxicities on normal tissues have been long-time needed. Previous studies reported that alteration in the expression and activity of growth factor receptors could not only directly perturb growth regulation, but also affect the sensitivity of cancer cells to various cytotoxic treatments, including RT [28,29]. Several groups identified that EGFR inhibitors could improve RT response and local control of human tumors, providing a kind of additional agents in anti-cancer therapy [10,11].

Nimotuzumab is a humanized IgG1 isotype monoclonal antibody of EGFR, which requires bivalent binding (i.e., binding with both antibody arms to two targets simultaneously) for stable attachment to cellular surface. Recently, it was reported that Nimotuzumab could substantially improve the radiosensitivity of brain tumor [30] and non-small cell lung cancer [31] cells with high or moderate levels of EGFR. In the present study, we determined to investigate the impact of Nimotuzumab on ESCC RT response and underling mechanisms. Our results demonstrated that although Nimotuzumab alone failed to inhibit ESCC cells growth, we did observe as well that Nimotuzumab dramatically enhanced radiation response of ESCC KYSE30 cell line (the cells with overexpression of EGFR) both *in vitro* and *in vivo*, as evidenced by increased radiation-inhibited cell growth and colony formation and radiation-mediated apoptosis. But similar results were not found in EGFR low-expressed ESCC cell line TE-1. These results revealed that Nimotuzumab only improved the radiosensitivity of ESCC cells with high expression of EGFR, suggesting a potential EGFR-dependent synergistic cytotoxicity of Nimotuzumab when combined with RT.

Previous reports documented that the anti-tumor effect of certain EGFR-specific mAbs is due to inhibition of ligand binding to EGFR and consequent inhibition of EGFR activation [32,33]. Nimotuzumab was identified only selectively binds to tumor cells that express moderate to high levels of EGFR [18]. To investigate the potential mechanisms of Nimotuzumab on promoting radiosensitivity of ESCC cells with high-level of EGFR, we first examined expression levels of EGFR and phosphorylated EGFR (i.e., p-EGFR, an active EGFR) before and after the administration of Nimotuzumab in ESCC cells. Our results showed that Nimotuzumab treatment alone did not result in any changes of the total EGFR levels in both EGFR overexpressed KYSE30 and EGFR low-expressed TE-1 ESCC cell lines. However, Nimotuzumab could down-regulate the levels of p-EGFR in KYSE30 cells. In TE-1 cells, in which the basic levels of EGFR were low and p-EGFR was not detected, the effect of p-EGFR downregulation cannot be observed in cells treated with Nimotuzumab. Furthermore, during the stimulation of EGF in both cells, we examined that the EGF-induced levels of p-EGFR in KYSE30 cells were dramatically inhibited by Nimotuzumab in a dose-dependent manner. But this down-regulation effect of p-EGFR by Nimotuzumab was not observed in TE-1 cell line. These observations clearly demonstrated that Nimotuzumab blocked the activation of EGFR in EGFR-overexpressed ESCC cells. Since previous studies documented that EGF-induced EGFR activation could decrease IGFBP-3 protein levels in ESCC TE-2 and TE-7 cell lines [27] and IGFBP-3 has been suggested as a key marker of radiosensitivity that enhances the susceptibility of ESCC to RT [25], in our study, we further examined the levels of IGFBP-3 in EGF-treated KYSE30 and TE-1 cell lines before and after Nimotuzumab administration. As anticipated, the treatment of Nimotuzumab could up-regulated the levels of IGFBP-3 in EGF-treated KYSE30 cells, concurrent with the decreased level of p-EGFR both *in vitro* and *in vivo*. Also, this phenomenon was not observed in EGF-treated TE-1 cell line. These data, collectively, suggested that Nimotuzumab might up-regulates IGFBP-3 expression in EGFR-overexpressed ESCC cells by suppressing the activation of EGFR.

IGFBP-3, a member of the IGFBP family, is the major carrier protein for insulin-like growth factors (IGF)-I or IGF-II in circulation [34,35]. Depending on the experimental context, IGFBP-3 has been shown to possess growth-stimulatory, antiproliferative, or proapoptotic activities *in vitro*[36,37]. In addition, IGFBP-3 has also been identified as a gene that is most highly up-regulated in EGFR-overexpressing esophageal cancer cell lines and primary esophageal tumors [26]. In our previous work, we have demonstrated that IGFBP-3 upregulation after Nimotuzumab administration involved in ESCC cells chemosensitivity to DDP [20]. To determine if IGFBP-3 participates in Nimotuzumab-enhanced radiosensitivity of ESCC cells with high expression of EGFR, we first knocked down the levels of IGFBP-3 in KYSE30 cells by specific shRNA against *IGFBP-3* gene and found that silence of IGFBP-3 dramatically reduced ESCC cell radiosensitivity. Furthermore, even after the treatment of Nimotuzumab, the radiosensitivity of IGFBP-3-silenced KYSE30 cells was almost not enhanced. These results provided evidences that the level of IGFBP-3 in EGFR-overexpressing ESCC cells is responsible, at least in part, for the increased radiosensitivity by Nimotuzumab. Krause and colleagues previously suggested that IGFBP-3 is a novel EGFR downstream target molecule in primary and immortalized human esophageal epithelial cells, and moreover, EGF could suppress IGFBP-3 expression through activation of MAPK in an EGFR-tyrosine kinase-dependent manner [27]. These data, taken together, prompt us to hypothesize that Nimotuzumab might up-regulate IGFBP-3 expression through EGFR-dependent pathway to enhance the radiosensitivity of ESCC cells. Clearly, further works are needed to clarify the regulation mechanisms of IGFBP-3 in ESCC and confirm our hypothesis in detail.

It is well known that, in the United States and Western countries, there has been a dramatic rise in the incidence of esophageal adenocarcinoma to equal or exceed the incidence of ESCC [38]. Therefore, one limitation of the current study is no esophageal adenocarcinoma cell lines are investigated. Another limitation of this study is only 2 ESCC cell lines are studied. In order to minimize the bias on the study results, we have taken a series of effective measures such as a panel of EGFR high and EGFR low ESCC cell lines and adenocarcinoma cell lines used in further study.

## Conclusions

In summary, ours results demonstrate, for the first time, that Nimotuzumab enhanced the anti-tumor efficacy of radiation in EGFR high-expression ESCC cells both *in vitro* and *in vivo*. Mechanism studies, as provided in this report, suggest that the enhanced radiotherapy effect by Nimotuzumab in EGFR high-expression ESCC cells might be dependent on the up-regulated expression of IGFBP-3 through EGFR-dependent pathway.

## Abbreviations

EGFR: Epidermal growth factor receptor; ESCC: Esophageal squamous cell carcinoma; RT: Radiotherapy; IGFBP-3: Insulin-Like growth factor binding protein-3; mAb: Monoclonal antibody; FISH: Fluorescent in situ hybridization; DMF_10_: Dose-modifying factor at a 10% survival level; FACS: Fluorescence activated cell sorting.

## Competing interest

The authors declare no conflict of interest.

## Authors' contributions

MZL and DX designed the study, carried out the data analysis, manuscript preparation. LZ carried out the data analysis. LRH carried out the data analysis. MX carried out the data analysis. MYC carried out the data analysis and IHC analysis. JXS and QQL carried out the statistical evaluation. YJL carried out the statistical evaluation. DQ carried out the statistical evaluation. ZZF carried out the data analysis. YXZ participated in the data discussion and manuscript preparation. All authors read and approved the final manuscript.

## Supplementary Material

Additional file 1 **Figure S1. **KYSE30 and TE-1 cells were plated in 96-well plates at a density of 2,000-3,000cells/well and incubated for 72 hours with indicated concentrations (6.25 μg/mL, 12.5 μg/mL, 25 μg/mL, 50 μg/mL, 100 μg/mL, 200 μg/mL) of Nimotuzumab. The viable cells were examined by MTT assay. Click here for file

Additional file 2 **Figure S2. **The percentages of viable ESCC cells cultured with Nimotuzumab or combination with RT at the indicated concentrations for 72 hours by MTT assay. Combination of RT and Nimotuzumab resulted in a significant higher level of cell death in KYSE30 cells (*P*=0.01; A), but not significant in TE-1 cells (*P*=0.087; B) Click here for file

Additional file 3 **Figure S3. **KYSE30 and TE-1 cells were treated with RT (2Gy, 10Gy) in presence or absence of Nimotuzumab (100 μg/mL) for 48 hours. The control, RT and combination treatment groups were collected for staining with annexin-V/PI to examine apoptosis rates. The samples with a statistically significant difference compared with the groups as indicated in KYSE30 cells. The experiments were performed in triplicate. Click here for file

Additional file 4 **Figure S4. **Knockdown of IGFBP-3 decreases sensitivity of KYSE30 cells to radiation. (**A**) IGFBP-3-silenced KYSE30 cells were incubated with or without Nimotuzumab (100 μg/mL) for 24 hours, and cultured in drug-free medium with 10% fetal bovine serum for 10–14 days. The colonies were stained and counted, and the result is representative at 6Gy of RT dosage. (**B**) KYSE30 cells survival were tested in 6 groups of control, control scrambled ShRNA, ShRNA, RT, ShRNA+RT and ShRNA+RT+Nimotuzumab by MTT assay. Click here for file

Additional file 5 **Figure S5. **(**A**) *In vitro*, radiotherapy alone did not induce up-regulation of IGFBP-3 expression in KYSE30 cells. (**B**) Effects of Nimotuzumab on the response of TE-1 ESCC xenografts to RT. TE-1 cells were injected subcutaneously in athymic nude mice. Treatment was initiated when tumors in each group (n=6) achieved an average volume of approximately 170–200 mm^3^. The mice were randomized into three groups: control, radiation alone (8Gy×1), and Nimotuzumab (a single dose of 1.0mg per mouse) combined with radiation. Tumor volume was determined at the indicated time points thereafter. Click here for file
