# Supplementary figures and images for: Nimotuzumab promotes radiosensitivity of EGFR-overexpression esophageal squamous cell carcinoma cells by upregulating IGFBP-3
Source: J Transl Med. 2012 Dec 11;10:249. doi: 10.1186/1479-5876-10-249 (PMC3540017; doi:10.1186/1479-5876-10-249)

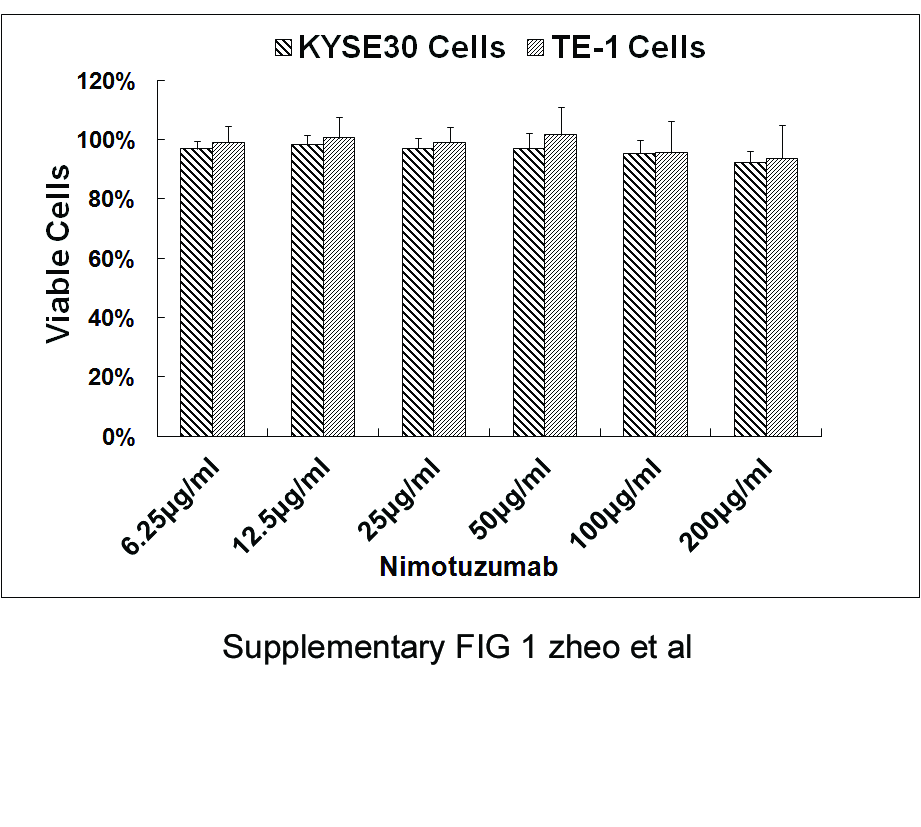

Supplement: Additional file 1 — Figure S1. KYSE30 and TE-1 cells were plated in 96-well plates at a density of 2,000-3,000cells/well and incubated for 72 hours with indicated concentrations (6.25 μg/mL, 12.5 μg/mL, 25 μg/mL, 50 μg/mL, 100 μg/mL, 200 μg/mL) of Nimotuzumab. The viable cells were examined by MTT assay. [file 1479-5876-10-249-S1.tiff]

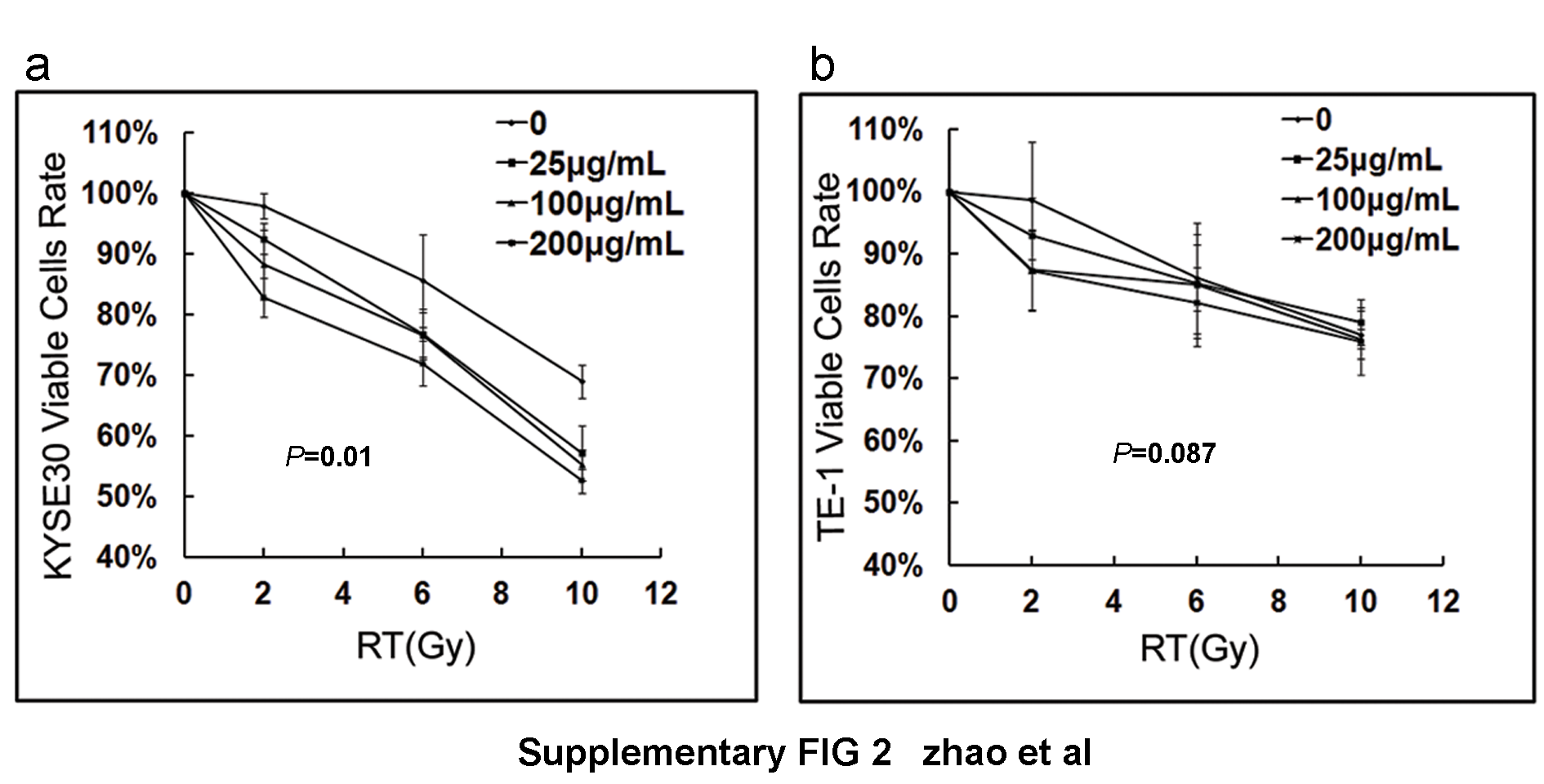

Supplement: Additional file 2 — Figure S2. The percentages of viable ESCC cells cultured with Nimotuzumab or combination with RT at the indicated concentrations for 72 hours by MTT assay. Combination of RT and Nimotuzumab resulted in a significant higher level of cell death in KYSE30 cells (P=0.01; A), but not significant in TE-1 cells (P=0.087; B) [file 1479-5876-10-249-S2.tiff]

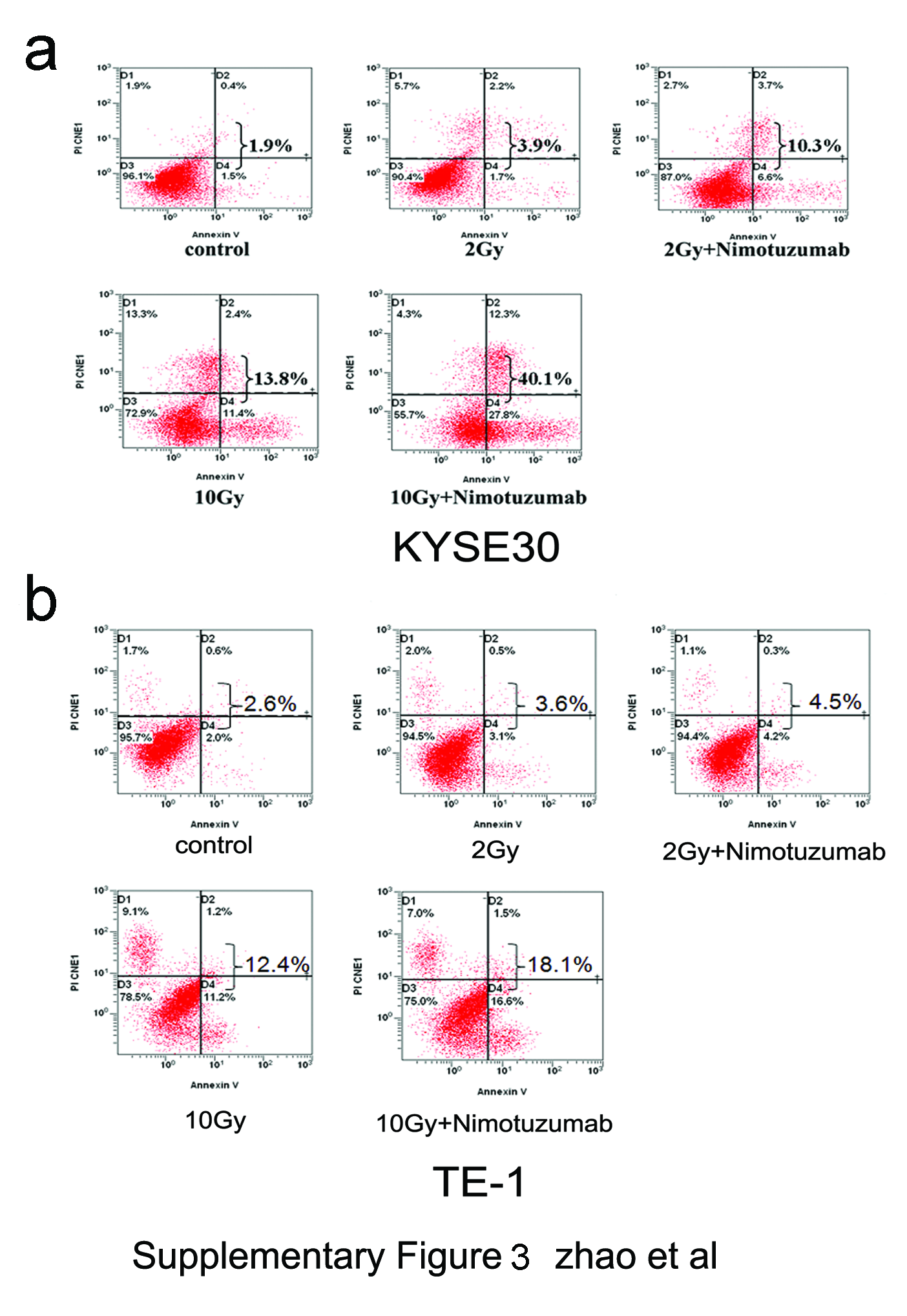

Supplement: Additional file 3 — Figure S3. KYSE30 and TE-1 cells were treated with RT (2Gy, 10Gy) in presence or absence of Nimotuzumab (100 μg/mL) for 48 hours. The control, RT and combination treatment groups were collected for staining with annexin-V/PI to examine apoptosis rates. The samples with a statistically significant difference compared with the groups as indicated in KYSE30 cells. The experiments were performed in triplicate. [file 1479-5876-10-249-S3.tiff]

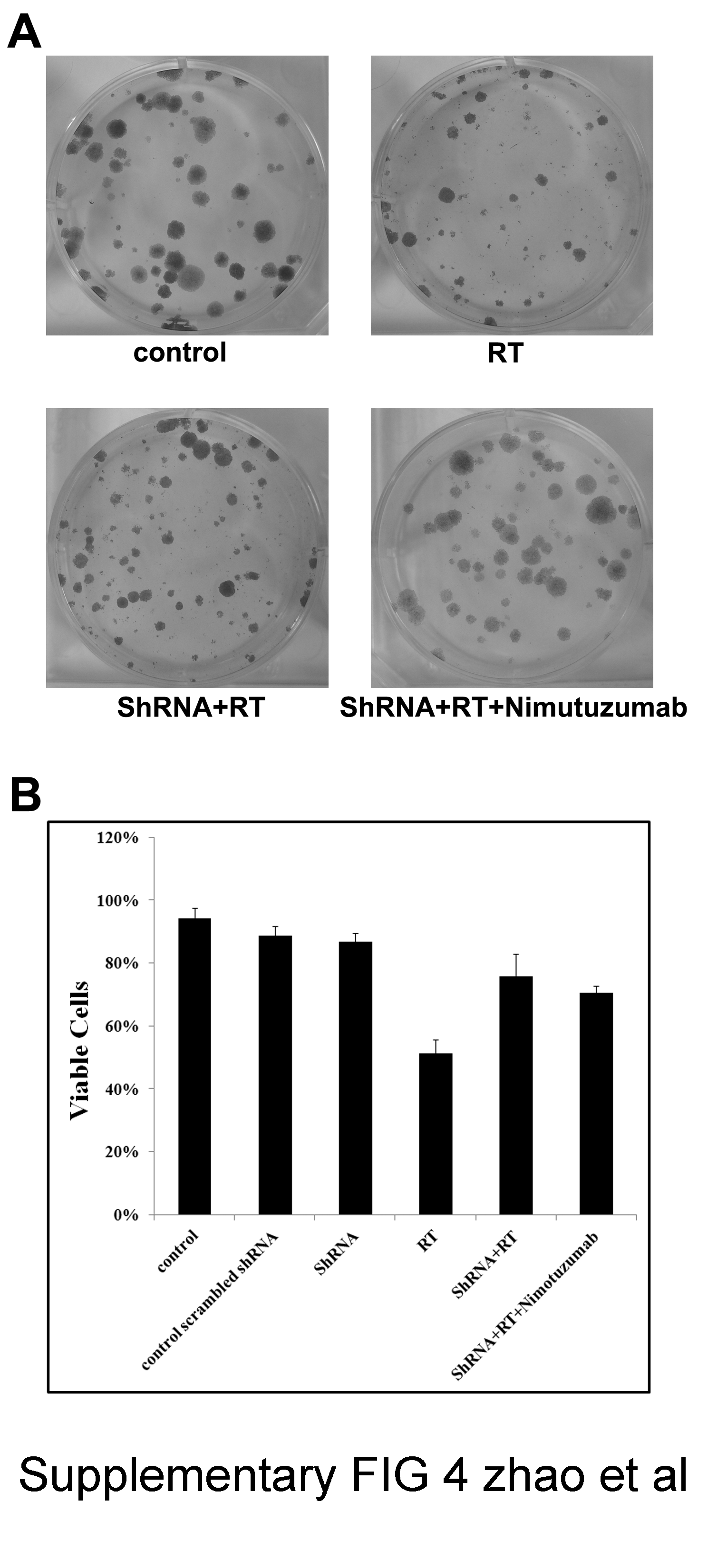

Supplement: Additional file 4 — Figure S4. Knockdown of IGFBP-3 decreases sensitivity of KYSE30 cells to radiation. (A) IGFBP-3-silenced KYSE30 cells were incubated with or without Nimotuzumab (100 μg/mL) for 24 hours, and cultured in drug-free medium with 10% fetal bovine serum for 10–14 days. The colonies were stained and counted, and the result is representative at 6Gy of RT dosage. (B) KYSE30 cells survival were tested in 6 groups of control, control scrambled ShRNA, ShRNA, RT, ShRNA+RT and ShRNA+RT+Nimotuzumab by MTT assay. [file 1479-5876-10-249-S4.tiff]

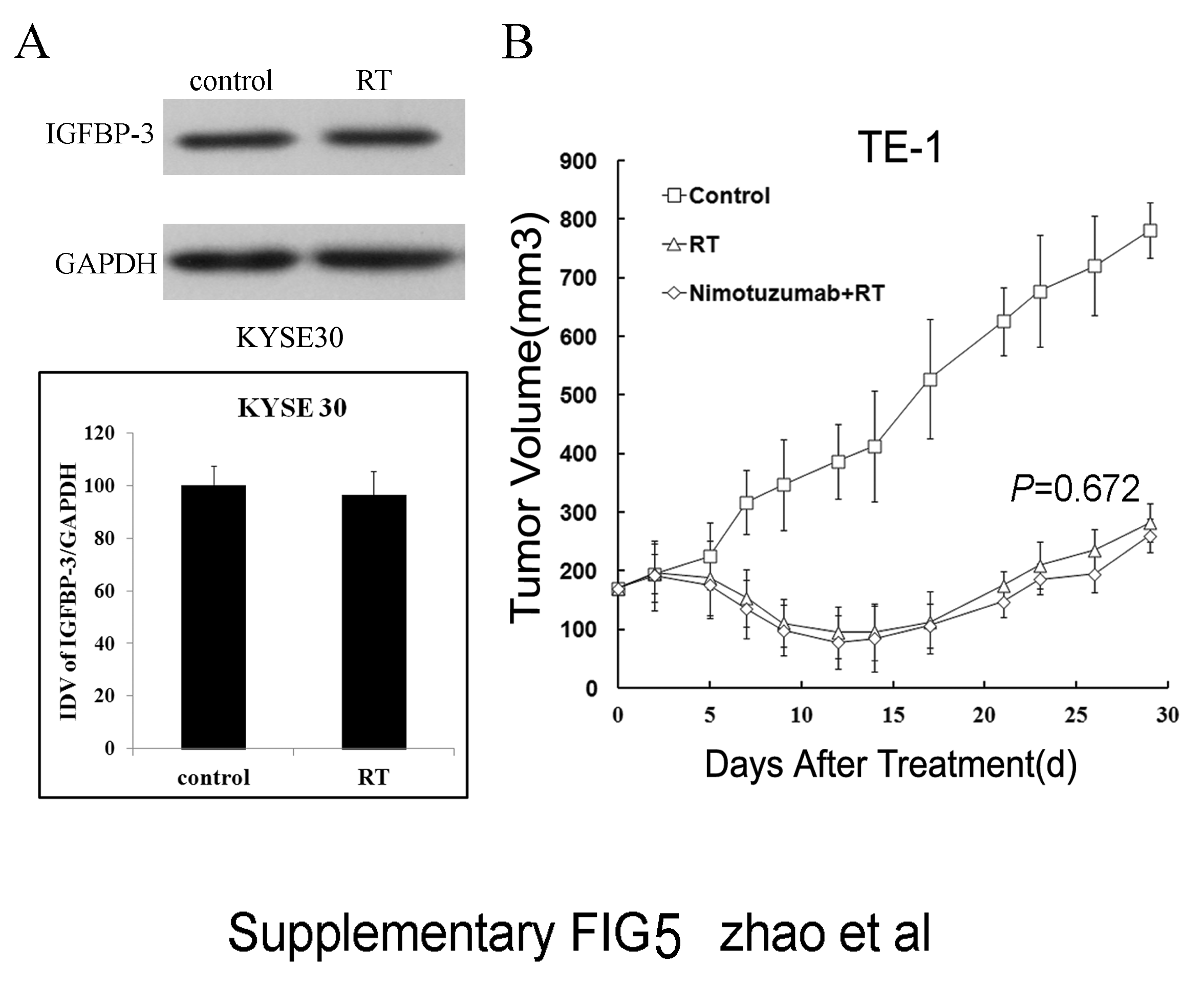

Supplement: Additional file 5 — Figure S5. (A) In vitro, radiotherapy alone did not induce up-regulation of IGFBP-3 expression in KYSE30 cells. (B) Effects of Nimotuzumab on the response of TE-1 ESCC xenografts to RT. TE-1 cells were injected subcutaneously in athymic nude mice. Treatment was initiated when tumors in each group (n=6) achieved an average volume of approximately 170–200 mm3. The mice were randomized into three groups: control, radiation alone (8Gy×1), and Nimotuzumab (a single dose of 1.0mg per mouse) combined with radiation. Tumor volume was determined at the indicated time points thereafter. [file 1479-5876-10-249-S5.tiff]
